# Supplementary figures and images for: Generating gnotobiotic bivalves: a new method on Manila clam (Ruditapes philippinarum)
Source: Microbiol Spectr. 2025 Aug 14;13(10):e01189-24. doi: 10.1128/spectrum.01189-24 (PMC12506633; doi:10.1128/spectrum.01189-24)

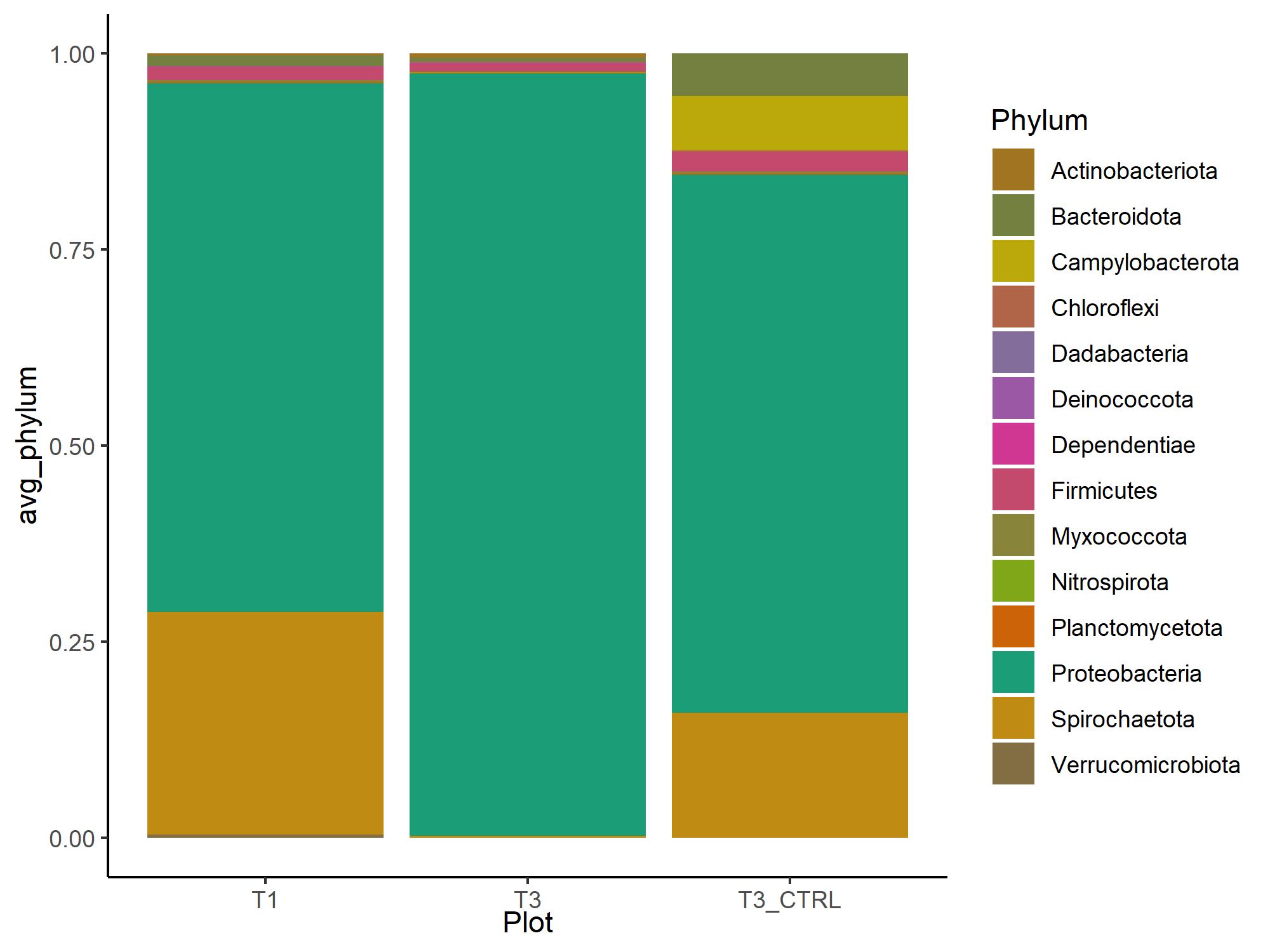

Supplement: Fig. S1 — Taxonomic composition of relative microbiome abundance at phylum level of acclimated clams (T1), antibiotic treated (T3-GF), and control clams (no antibiotic treatment; T3-Control). [file spectrum.01189-24-s0001.png]

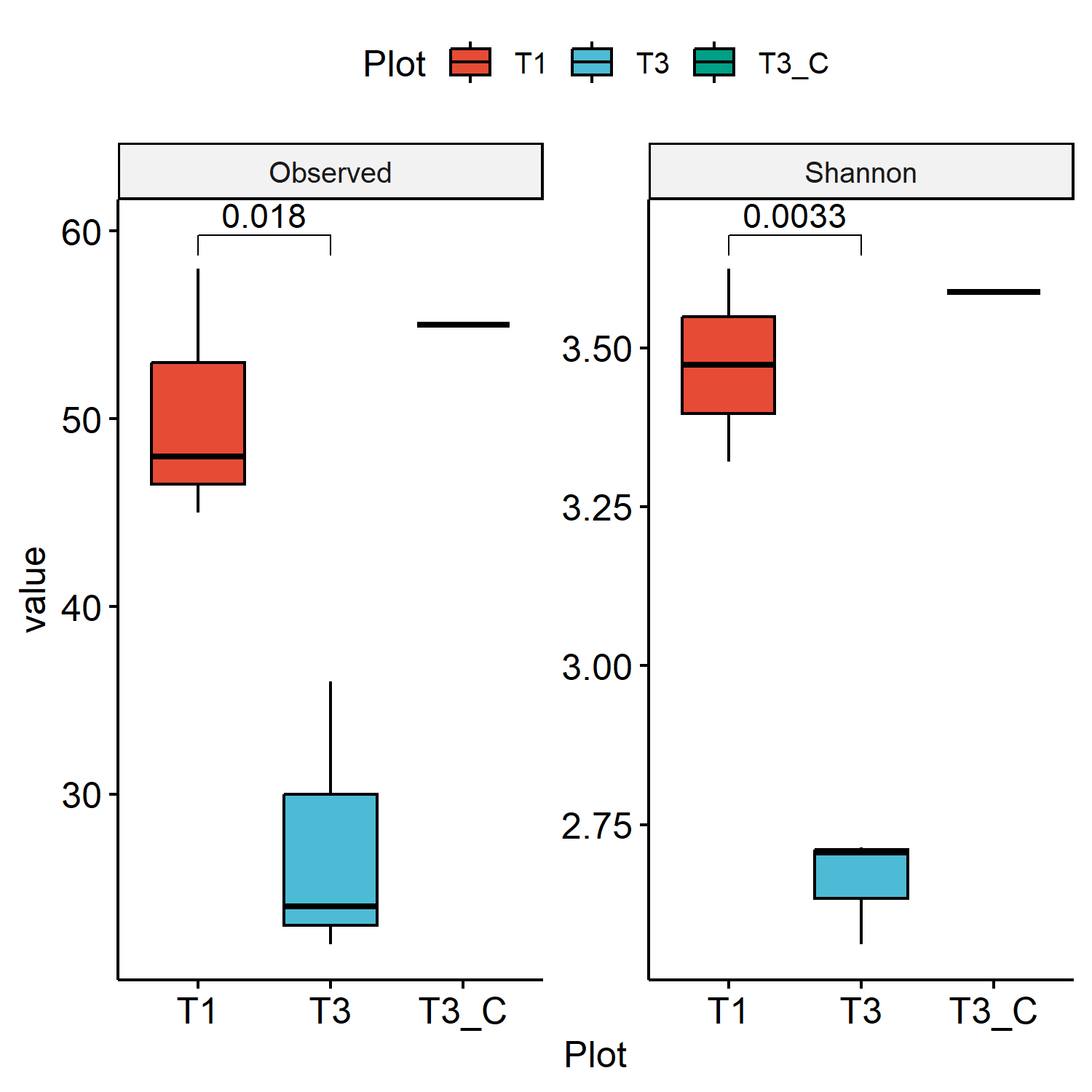

Supplement: Fig. S2 — Observed and Shannon indices were estimated for the two time-points. [file spectrum.01189-24-s0002.tif]

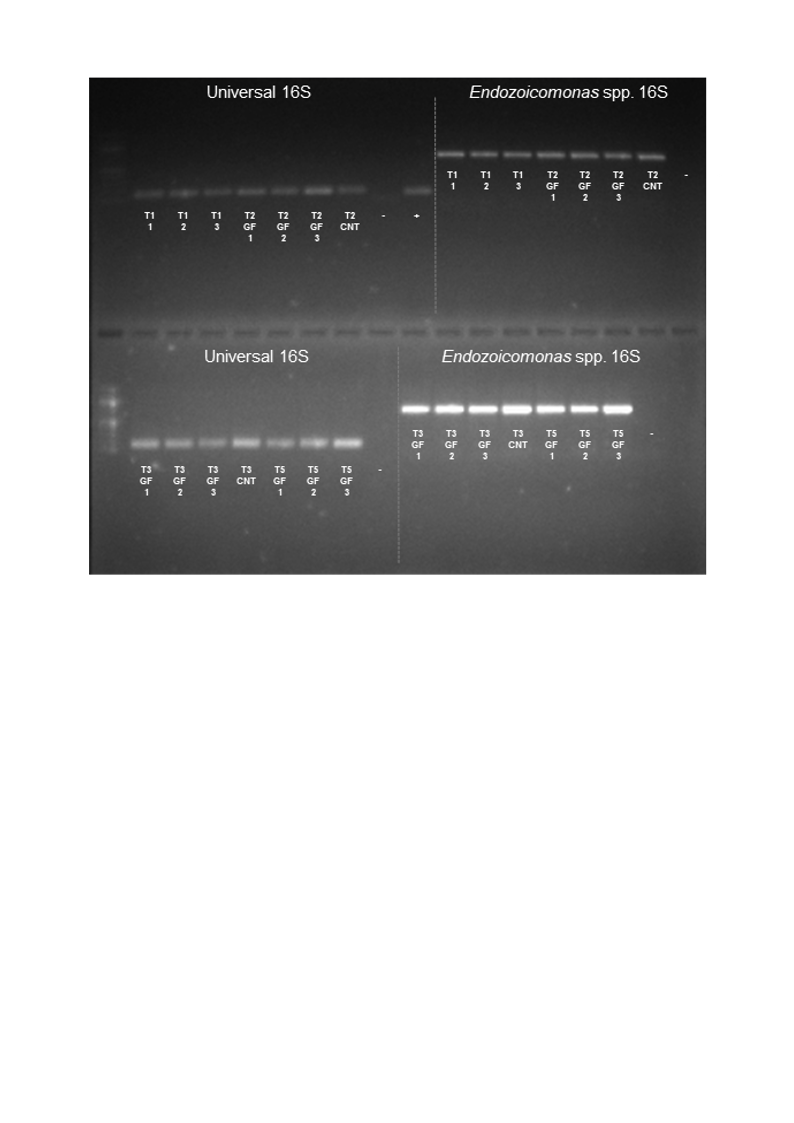

Supplement: Fig S3 — 1.5% agarose gel showing end-point PCR amplification products from experimental samples and controls. [file spectrum.01189-24-s0003.png]

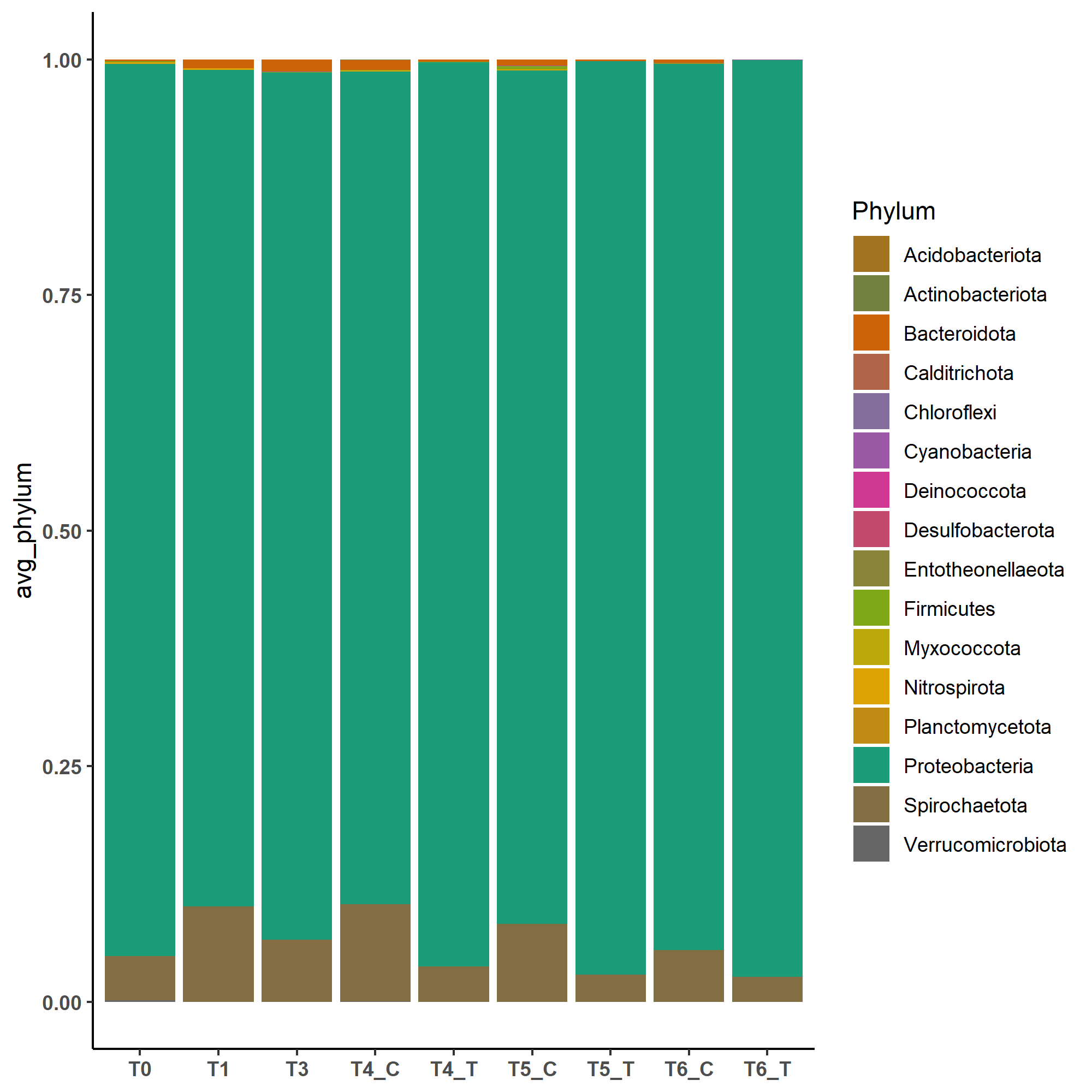

Supplement: Fig. S4 — Taxonomic composition of relative microbiome abundance of experimental samples at phylum level. [file spectrum.01189-24-s0004.png]
