# Supplementary material for: Generating gnotobiotic bivalves: a new method on Manila clam (Ruditapes philippinarum)
Source: Microbiol Spectr. 2025 Aug 14;13(10):e01189-24. doi: 10.1128/spectrum.01189-24 (PMC12506633; doi:10.1128/spectrum.01189-24)
Supplement: Supplemental material — Supplemental figure legends. [file spectrum.01189-24-s0005.docx]

**Supplementary Figure Legends**

**Fig. S1** Taxonomic composition of relative microbiome abundance at phylum level of acclimated clams (T1), antibiotic treated (T3 - GF) and control clams (no antibiotic treatment; T3 - Control).

**Fig. S2** Observed and Shannon indices were estimated for the two time-points. P-values estimated through t.test analysis are reported between T1 and T3, highlighting a significant result for both indices.

Fig. S3 1.5% agarose gel showing end-point PCR amplification products from experimental samples and controls, using primers targeting the universal 16S rRNA gene and the 16S rRNA gene of Endozoicomonas spp., respectively. Sample abbreviations: T1 – acclimated clams; T2 – 6 hours post-antibiotic administration; T3 – 20 hours post-antibiotic administration; T5 – 6 hours post-transplant of the mock community; GF – antibiotic-treated clams; CNT – untreated (control) clams; + positive control; - negative control.

**Fig. S4** Taxonomic composition of relative microbiome abundance of experimental samples at phylum level.
