# Supplementary material for: Generating gnotobiotic bivalves: a new method on Manila clam (Ruditapes philippinarum)
Source: Microbiol Spectr. 2025 Aug 14;13(10):e01189-24. doi: 10.1128/spectrum.01189-24 (PMC12506633; doi:10.1128/spectrum.01189-24)
Supplement: Table S2 — Sanger sequence of 16S rRNA gene of Endozoicomonas spp. detected in antibiotic-treated clams. [file spectrum.01189-24-s0007.docx]

**Table S2.** Sanger sequence of 16S rRNA gene of *Endozoicomonas* spp. detected in antibiotic-treated clams.

| **Sample** | **Sequence** |
| --- | --- |
| T3-GF | AGGCCTAACCATGCAAGTCGAGCGGTAACAGGAGGAAGCTTGCTTTCTGCTGACGAGCGGCGGACGGGTGCGTAACACGTAGGAATCTGCCCGGTAGTGGGGGATAGCCCGGAGAAATCCGGATTAATACCGCATACGTCCTAAGGGGGAAAGCAGGGGATCTTCGGACCTTGCGCTATCGGATGAGCCTGCGTCGGATTAGCTAGTTGGTGGGGTAAAGGCCTACCAAGGCCACGATCCGTAGCTGGTCTGAGAGGATGATCAGCCACACTGGGACTGAGACACGGCCCAGACTCCTACGGGAGGCAGCAGTGGGGAATATTGCACAATGGGGGAAACCCTGATGCAGCCATGCCGCGTGTGTGAAGAAGGCTCTAGGGTTGTAAAGCACTTTCAGTAGGGAGGAAAGGGTGGAGGTTAATACCCGTCATCTGTGACGTTACCTACAGAAGAAGCACCGGCTAACTCCGTGCCAGCAGCCGCGGTAATACGGAGGGTGCAAGCGTTAATCGGAATTACTGGGCGTAAAGAGTACGTAGGCGGCTGCCTAAGTTGGATGTGAAAGCCCTGGGCTTAACCTGGGAACTGCATCCAAAACTGGGCGGCTAGAGTGCGGAAGAGGAGTGTGGAATTTCCTGTGTAGCG |
