# Supplementary material for: Generating gnotobiotic bivalves: a new method on Manila clam (Ruditapes philippinarum)
Source: Microbiol Spectr. 2025 Aug 14;13(10):e01189-24. doi: 10.1128/spectrum.01189-24 (PMC12506633; doi:10.1128/spectrum.01189-24)
Supplement: Table S3 — Statistical results showing p-values and adjusted p-values from ANOVA tests assessing variance in species abundance over time. [file spectrum.01189-24-s0008.docx]

**Table S3.** Statistical results showing p-values and adjusted p-values (p.adj) from ANOVA tests assessing variance in species abundance over time. Anova tests were performed using the compare_means function in the ggpubr package.

| **Species** | **p-value** | **p.adj** |
| --- | --- | --- |
| Halomonas alkaliphila | 0.00117 | 0.00035 |
| Shewanella colwelliana | 0.000530 | 0.0011 |
| Vibrio diazotrophicus | 0.00114 | 0.0011 |
